# Using the Robson Classification to Explain the Fluctuations in Cesarean Section

**DOI:** 10.1155/2020/2793296

**Published:** 2020-11-12

**Authors:** H. Cammu, E. Martens, G. Van Maele

**Affiliations:** ^1^Study Centre for Perinatal Epidemiology & Vrije Universiteit Brussel, Laarbeeklaan 101 Brussels 1090, Belgium; ^2^MSPT Study Centre for Perinatal Epidemiology, Koning Albert II laan 35 Brussels 1030, Belgium; ^3^Department Medical Statistics Ghent University Belgium, C. Heymanslaan 10, Ghent 9000, Belgium

## Abstract

**Purpose:**

As the rate of cesarean sections (CS) continues to rise in Flanders (northern part of Belgium), it is important to understand the reasons behind this evolution and to find ways to achieve appropriate CS rates. For this analysis, we categorized CS changes between 1992 and 2016, applying the Robson 10-Group Classification System (TGCS). We also applied the TGCS to analyze the information of the only clinics where between 2008 and 2016, the absolute CS rate had fallen by more than two percent.

**Methods:**

This paper is based on a population-based cross-sectional study. Robson's TGCS was used to analyze CS rates for the years 1992, 2000, 2008, and 2016, using the Flemish population-based birth register.

**Results:**

Between 1992 and 2016, the overall CS rate increased from 11.8% in 1992 to 20.9% in 2016. The major contributors to that increase were (a) single, cephalic nulliparous women, at term in spontaneous labor (Robson group 1); (b) single, cephalic nulliparous women, at term in induced labor or CS before labor (group 2); and (c) multiparous women with single cephalic at term pregnancy with history of CS (group 5). In the subgroup of the seven clinics where the collective CS rate had decreased from 23.2% in 2008 to 19.3% in 2016, the main contributors to this decrease were Robson groups 1 and 2.

**Conclusions:**

The CS increase in Flanders between 1992 and 2016 is mainly the result of the absolute CS increase in the childbirth of nulliparous women with a single cephalic baby at term in spontaneous or induced labor and in women with a single cephalic presentation at term and a previous CS. Further research in these aforementioned groups is needed to identify the real reasons for the CS increase.

## 1. Introduction

For more than two decades, CS births easily rise beyond 25% in most middle- and high-income countries, whereas in low-income countries, women often do not have access to this method of birth even when it is needed. [1, 2]. The WHO, in an 8-9 October 2014 meeting in Geneva, concluded that there is no reason to change the guideline of 1985, stipulating that, at population level, CS rates higher than 10% were not associated with reductions in maternal and newborn mortality rates provided that the fetus was alive at the beginning of labor [3–7].

The CS analysis has been hindered by the lack of a consistent classification system to monitor and compare CS rates. To address this lack, Robson proposed the 10-Group Classification in 2001 [8]. Ten years later (2011), the WHO proposed the adoption of the Robson “10-Group Classification System (TGCS)” which allows for comparison and analysis of CS rates within and between different facilities and across countries, regions, and time. The TGCS categories are based on five routinely collected characteristics such as gestational age, parity, fetal presentation, multiple births, and previous cesarean section, and they clearly distinguish between prelabor CS and those after spontaneous and induced labor (Table 1). The classification is simple, reproducible, mutually exclusive, and clinically relevant: every delivering woman can be classified into one of the ten groups which allows comparison and analysis of CS rates. This Robson 10-Group Classification for cesarean section has now been endorsed by the WHO, the International Federation of Gynecology and Obstetrics (FIGO), and the European Board and College of Obstetrics and Gynecology (EBCOG) and is used in many countries [9, 10].

The aim of the study was twofold. First, we wanted to analyze the 25-year evolution (1992-2016) of CS in Flanders by using the aforementioned Robson 10-Group Classification System. To that end, we compared the years 1992-2000-2008-2016. Second, we wanted to find out whether the TGCS groups that contributed most to a CS increase between 1992 and 2016 would be the same as those that would be responsible for the CS decrease. For this reason, we applied the Robson Classification System to those maternity clinics where between 2008 and 2016, the absolute CS rate had dropped by more than two percent.

## 2. Materials and Methods

Flanders, the Dutch-speaking northern region (13,522 km^2^) of the federal state of Belgium, has 6.3 million inhabitants and on average 65,000 births per year of which >99% take place in hospital maternity units. Flanders is an affluent society with a universal and equitable access to maternity care covered by compulsory health insurance for everyone and highly urbanized. Distance to the 68 maternity units is not an issue. Perinatal data from all maternity units are centrally collated by the Study Center for Perinatal Epidemiology (SPE), an independent, regionally funded, center that registers all births of >500 grams and/or 22-week gestation. The SPE refers to fetal death as to a stillborn baby and infant mortality as the sum of (i) early neonatal (0-6 days-23 h-59 min), (ii) late neonatal (7-27days-23 h-59 min), and (iii) postneonatal (28-365 days) deaths. The TGCS was applied to analyze this 25-year evolution (1992-2016). The 1992 TGCS was incomplete because the data item “history of a previous CS” was only included from 2000 onwards. We first determined the relative size, in percent, of each Robson group, for the years 1992/2000/2008 and 2016. Thereafter, we calculated the CS rate in each group for the aforementioned years. Then, we determined the absolute contribution of CS by a particular group to the overall CS rate. We then analyzed in more detail within the TGCS the following: mothers' age (with an analytical threshold at 35 years), whether or not artificial reproduction technology (ART) was used, and fetal weight (which we set at a minimum of 4000 g). Finally, we applied the TGCS to the seven maternity clinics where between 2008 and 2016, the absolute SC rate had dropped by more than two percent.

### 2.1. Statistical Analysis

We performed our analysis with the statistical software language R (R version 3.5.2 (2018-12-20) [11]. Categorical data are expressed as frequencies with percentages; continuous data are expressed as mean (standard deviation). The comparison between the selected years has been carried out using a test for proportions. The Fisher exact test with odds ratio and 95% confidence intervals is used in situations with two groups. Effects of impacts of other variables on the odds ratio were analyzed with logistic regression. The significance level has been set at *α* = 0.05. Ethical approval for analysis of the data was obtained.

## 3. Results

Some patients and pregnancy characteristics changed over 25 years (Table 2). Women are now older when they give birth for the first time compared to 1992. As a result, patients have to rely more on ART. The incidence of labor induction decreased modestly, especially in multiparous women. Preterm birth (<37 weeks) increased between 1992 and 2000 and then leveled off. Other noticeable changes between 1992 and 2016 were the dramatic increase of epidural analgesia and the gradual decline in instrumental deliveries. Between 1992 and 2016, the absolute CS rate nearly doubled in Flanders (Table 2). The relative size of each of the TGCS in total birth changed over time but not to a clinically relevant degree except for a pertinent increase in women with a previous CS (Robson group 5) (Table 3). There was a gradual increase in absolute CS in each of the ten groups throughout the 25 years (Table 3). The largest CS increase per group was seen in nulliparous, singleton, at term women in spontaneous or induced labor (Robson group 1 and 2a) (Table 4). The most important absolute contribution to the overall CS rate, however, was from multiparous, singleton at term women with a baby in cephalic presentation and a previous CS (Robson group 5) (Table 5). We also noticed a CS increase in groups 6-10. However, groups 6-10 accounted for only 10 (1992) to 12.1% (2016) of the total obstetric population. The contribution of groups 6-10 to the overall CS rate did not differ much over the years and remained small but stable at around a third of all cesareans (35% in 1992, 37% in 2000, 35% in 2008, and 33% in 2016). The sum of the relative size of groups 1 and 2 did not change significantly throughout the years: the sum of groups 1 and 2 amounted to 40.3% in 1992, 39.6% in 2000, 40.1% in 2008, and 38.4% in 2016 (Tables 5 and 3). Taking into account that the incidence of breech presentation (groups 6 and 7) as a medical driver for CS is minimal, we conclude that the global increase in CS percentage in Flanders was mainly due to the CS increase in nulliparous women with a single cephalic baby at term in spontaneous or induced labor and in women with a single cephalic presentation at term and a previous CS.

We applied the TGCS to nulliparous women aged at or above 35 years, to nulliparous women pregnant after ART, and to nulliparous women who delivered a baby of 4 kg or more. The absolute CS rate in women at or above 35 years doubled from 14.3% (1992) over 20% (2000) and 25.3% (2008) to 28.8% (2016). The main contributors to this CS increase were groups 1 and 5. Absolute CS rates after ART increased from 21.1% (1992) over 24.9% (2000) and 29.1% (2008) to 30.5% (2016). The main contributor to this CS increase was group 1. The absolute CS rate in nulliparous women who delivered a baby of 4 kg or more increased from 12.2% over 16.8% and 18.4% to 21.4%. Here, the main contributors to the increase were groups 1 and 2.

Between 2008 and 2016, absolute CS rates decreased by more than two percent in seven peripheral maternity clinics (Tables 6, 7, and 8). In 2008, the mean CS rate in these seven clinics amounted to 23.2% (1127 SC/4857 women). Eight years later, CS rates had dropped to 19.3% (895 SC/4627 women). The relative size of each of the TGCS in total birth between 2008 and 2016 only showed a significant decrease in induced labor and in prelabor CS in nulliparous women (Table 7). In 2016, spontaneous labor among singleton, cephalic, at term women less often led to a CS compared to 2008 (Table 8). The decline in CS rate in Robson groups 1, 2, and 5 was responsible for 82% of the fall in CS (Table 6).

Between 1992 and 2016, a slight fall in stillbirth but a notable fall in early neonatal and infant death was observed (Table 2). The decrease was mainly noticed between 1992 and 2000 after which the effect slowed down. Early neonatal death in Robson groups 1 and 2 was very low: respectively, 0.10% (*N* = 18) and 0.16% (*N* = 14) in 1992, 0.07% (*N* = 10) and 0.10% (*N* = 9) in 2000, 0.05% (*N* = 9) and 0.07% (*N* = 6) in 2008, and 0.03% (*N* = 3) and 0.07% (*N* = 6) in 2016.

## 4. Discussion

In Flanders, there is a significant increase in the absolute CS rate between 1992 and 2016. The main reason for the latter was the CS increase in at term nulliparous women with a singleton in cephalic presentation after spontaneous or induced labor (Robson groups 1 and 2). However, women with a previous CS (group 5) represent the largest single contributors to the overall CS rate. This higher contribution was mainly due to the bigger size of the group. Indeed, the incidence of vaginal birth after CS in Flanders did not change significantly over the years (Table 7) but the number of women with a CS scar did (Table 5). Induction of labor and birth weight of 4 kg or more showed the opposite. In this case, the size of the groups barely changed (there was even a slight decrease in labor induction). However, women whose labor was induced and women with a baby of 4 kg or more much more often underwent a CS (Table 2). When ART techniques were used and when maternal age was at or above 35 years, both absolute size and percentage mattered: ART and age at or above 35 years increased in absolute size but the SC percentage for both components also increased.

The CS decrease between 2008 and 2016 that was seen in some maternity clinics was mainly due to the decrease in CS in Robson group 2 and also in groups 1 and 5. An Irish study [12] showed exactly what we found. In 20 out of 21 countries [13] with a mixed Human Development Index, CS rates increased over time: nulliparous women (Robson groups 1 and 2) were the single largest relative contributor to the overall CS rate, accounting for about a third of all CS rates, followed by women who had previously had a CS (group 5) who accounted for a quarter of the rates. In Japan, a small decrease in CS rates over time was mainly due to a decline in CS rates in Robson groups 1 and 2 [13]. In 2012, the CS rate of 19 Lithuanian hospitals, in which TGCS was introduced, was 26.9%. Two years later, the CS rate in these hospitals was significantly decreased to 22.7%. The greatest decrease was detected in group 2 [14]. This latter corroborated with our findings in the seven clinics with a CS decrease between 2008 and 2016. In an American study, those with a previous cesarean delivery (Robson group 5) were the largest contributor to the overall rate of cesarean delivery over the period 2005 through 2014 [15]. One may assume that by now, wherever the TGCS is used, one will always find the same groups responsible for the CS increase (Robson groups 1, 2, and 5).

The TGCS does not explain why CSs are done [16]. The TGCS is a common starting point for further analysis and not the endpoint of the CS debate [17]. If we want to improve the usefulness of the TGCS, we need to know more about the real reasons why a CS was performed. This more advanced application has already been occasionally done [18, 19]. However, there is actually no accepted international classification for CS indications. Therefore, we propose to standardize the indications for CS per Robson group. This will not be easy in population-based data (like ours), as even a simple question of whether or not there was a medical indication is often poorly ascertained. Moreover, indications are recorded far less reliable than the interventions themselves [20, 21].

Differences in outcome can depend on the quality of the data. According to Robson, the TGCS can be used to assess data quality [16]: group 9 (transverse or oblique lie) is a good marker to assess data quality because it is very consistent in size between 0.2% and 0.6% with a CS ratio of nearly 100%. Between 1992 and 2016, our group 9 had a relative size of 0.3% with >90% SC (Tables 3 and 4). We can therefore conclude that our database is of acceptable quality.

Differences in outcome may also be due to differences in patient population. Older maternal age and pregnancy after ART were associated with more CSs. This confirms previous Flemish [22, 23] and Danish [24] research. Differences may be the result of physician's practice style. Today, gynecologists are moving faster to a CS than two decades ago, but we do not know why.

Another element that might influence CS rates are childbirths where epidural analgesia was used. Between 1992 and 2000, an important epidural increase was seen. According to a Cochrane review (2011), women who use epidural analgesia are at increased risk of having an instrumental delivery. Epidural analgesia had no impact on the CS rate [25]. In Flanders, the opposite occurred: an important increase in epidural analgesia, an increase in CS, but a decrease in instrumental delivery. Whether these features are interrelated is impossible to ascertain.

The strength is that assessment and outcome are based on data from an entire population. The study reflects what happened in reality. The limitations were related to the incompleteness of the dataset: in 1992, “history of a previous CS” was not included in the set. Data related to indications for CS such as fetal distress or dystocia were incomplete and thus unreliable. We had no data on the percentage of CS due to maternal request or due to the impact of maternal illness. Strategies to reduce CS rates must also assess changes in perinatal outcome. We only included stillbirth and early neonatal and infant mortality. However, the retrospective, cross-sectional nature of the study did not allow us to determine a causal link between the CS rate and the perinatal outcome. Good data on neonatal and maternal morbidity was missing.

## 5. Conclusions

In Flanders, the CS rate increased from 11.8% to 20.9% between 1992 and 2016 and the TGCS allowed us to analyze that increase. The largest absolute increases in CS were seen in multiparous, singleton at term women with a previous CS (Robson group 5) and in nulliparous singleton, at term women in spontaneous or induced labor (Robson groups 1 and 2).

Between 1992 and 2016, gynecologists gradually proceeded more quickly to a CS when treating women at or above 35 years, after ART, after labor induction, or when the birth weight was 4 kg or more. The main contributor to this increase came from Robson group 1.

Maternity clinics that succeeded in reducing their absolute CS rate between 2008 and 16 did so because they were less likely to do a CS in nulliparous, singleton, cephalic, at term women in induced or in spontaneous labor (Robson groups 1 and 2). However, the TGCS does not allow us to explain by indication what the reasons for fluctuations in CS rate are. We need to be better at structured routine data collection. This will be the most difficult task.

## Figures and Tables

**Table 1:** The Robson 10-Group Classification System (TGCS) [13].

1	Nulliparous, singleton, cephalic, ≥37-week gestation, in spontaneous labor
2	Nulliparous, singleton, cephalic, ≥37-week gestation, induced (2a) or CS before (2b) labor
3	Multiparous (excluding previous CS), singleton, cephalic, ≥37-week gestation, in spontaneous labor
4	Multiparous (excluding previous CS), singleton, cephalic, ≥37-week gestation, induced (4a) or CS before (4b) labor
5	All multiparous women with a previous CS, singleton, cephalic, ≥37-week gestation
6	All nulliparous with a single breech pregnancy
7	All multiparous with a single breech pregnancy (including previous CS)
8	All multiple pregnancies (including previous CS)
9	All women with a single pregnancy in transverse or oblique lie (including previous CS)
10	All preterm singleton, cephalic, <37-week gestation pregnancies (including previous CS)

**Table 2:** Patient, pregnancy, and delivery characteristics.

	1992	2000	2008	2016
Number of women	67477	60987	68195	64323
Nulliparous women (%)	45.9	46.2	46.9	45.1
Mean age (y)	26.4	27.5	28.1	28.9
Age ≥ 35 y (%)	2.5	5.1	7.7	10.1
Age < 20 y (%)	3.8	4.3	3.7	2.3
Multiparous women (%)	54.1	53.8	53.1	54.9
Mean age (y)	29.6	30.7	31.1	31.6
Age ≥ 35 y (%)	9.6	15.8	20.1	22.4
Artificial reproduction technology (ART)				
Ovulation induction	1.4	1.7	2.1	2.6
IVF/ICSI (%)	0.8	1.7	3.3	4.6
Multiple pregnancy (%)	1.6	1.8	1.9	1.7
Preterm birth <37 w (%)	5.6	7.1	7.4	7.6
Preterm birth <32 w (%)	0.8	1.0	1.0	1.2
Epidural (%)	34.8	61.7	67.4	70.3
Labor induction (%) Po	27.8	30.4	25.9	25.6
Labor induction (%) Pn	28.4	30.1	24.8	21.8
Cesarean section (%)	11.8	16.4	19.5	20.9
Instrumental delivery (%)	14.5	12.4	10.4	9.8
Number of births	68613	62122	69466	65440
Birth weight				
<1500 g	0.9	1.1	1.1	1.2
1500-2499 g	5.2	5.7	5.8	5.7
2500-3999 g	85.1	85.0	84.1	84.3
4000-4499 g	7.8	7.2	7.9	7.8
≥4500 g	1.0	0.9	1.1	1.0
Stillbirth (%)	0.54	0.45	0,42	0,44
Early neonatal death (%)	0.30	0.23	0.20	0.18
Infant death (%)	0.66	0.46	0.39	0.37

*p* < 0.001.

**Table 3:** Robson's 10-Group Classification of Flemish women giving birth in 1992-2000-2008-2016: relative size of each group in total birth (%).

	1992 (%)	2000 (%)	2008 (%)	2016 (%)	*p* value
1	27.1	25.1	27.2	26.6	^∗^
2a	12	13.1	11.4	10.7	^∗^
2b	1.2	1.4	1.5	1.1	^∗^
3	ND	27.2	27.4	29.2	^∗^
4a	ND	14.6	11.7	10.7	^∗^
4b	ND	1.0	0.7	1.1	^∗^
5	ND	5.6	7.9	8.4	^∗^
6	2.3	2.7	2.8	2.5	^∗^
7	1.7	1.8	1.8	1.7	*p* = 0.12
8	1.6	1.8	1.9	1.8	*p* = 0.07
9	0.3	0.3	0.3	0.3	*p* = 0.02
10	4.1	5.4	5.5	5.8	^∗^

Robson 3, 4a, 4b, 5: *p* value comparison 2000-2008-2016. NA = no data. ^∗^*p* < 0.001.

**Table 4:** Robson's 10-Group Classification of Flemish women giving birth in 1992-2000-2008-2016: cesarean sections per group (%). ND = no data.

	1992 (%)	2000 (%)	2008 (%)	2016 (%)	92-16 CS
1	4.1	6.8	8.8	10.1	×2.5
2a	9.7	15.4	19.9	23.6	×2.4
2b	100	100	100	100	
3	ND	1.4	1.6	1.9	
4a	ND	2.4	2.9	3.9	
4b	100	100	100	100	
5	ND	62.4	62.8	66.5	
6	74.6	88.4	95.2	95.8	×1.3
7	51.2	68	84.5	87.9	×1.7
8	35.9	46.2	55.5	55.8	×1.6
9	95.4	94.0	95.5	98.6	
10	17.6	23.6	25.8	29,0	×1.6
Total CS	11.8%	16.4%	19.5%	20.9%	

**Table 5:** Robson's 10-Group Classification of Flemish women giving birth in 1992-2000-2008-2016: absolute contribution of cesarean sections per group to the total cesarean section rate. ND = no data.

	1992 (%)	2000 (%)	2008 (%)	2016 (%)	∆ SC 92-16
1	1.1	1.7	2.4	2.7	+1.6
2a	1.2	2.0	2.3	2.5	+1.3
2b	1.2	1.4	1.5	1.1	-0.1
3	ND	0.4	0.4	0.6	(+0.2)
4a	ND	0.3	0.3	0.4	(+0.1)
4b	ND	1	0.7	1.1	(+0.1)
5	ND	3.5	4.9	5.6	(+2.1**)**
6	1.7	2.4	2.6	2.4	+0.7
7	0.9	1.3	1.5	1.5	+0.6
8	0.6	0.8	1	1	+0.4
9	0.2	0.3	0.3	0.3	+0.1
10	0.7	1.3	1.5	1.7	+1
Total %	11.8%	16.4%	19.5%	20.9%	(): 2000-16

**Table 6:** Robson's 10-Group Classification of a selected group of Flemish women giving birth in 2008 and 2016: Cesarean section contribution per group (%).

	2008	2016	OR (95% CI)
1	3.31	2.53 (-0.78)	1.322 (1.03-1.70)
2a	2.94	2.05 (-0.89)	1.447 (1.10-1.90)
2b	2.08	1.15 (-0.93)	1.833 (1.30-2.61)
3	0.58	0.35 (-0.23)	1.671 (0.87-3.31)
4a	0.47	0.35 (-0.12)	1.371 (0.69-2.78)
4b	0.54	0.65 (+0.11)	0.825 (0.47-1.45)
5	6.42	5.81 (-0.61)	1.112 (0.94-1.32)
6	3.01	2.55 (-0.46)	1.184 (0.92-1.53)
7	1.56	1.69 (+0.13)	0.927 (0.67-1.29)
8	0.97	0.76 (-0.21)	1.282 (0.81-2.05)
9	0.19	0.22 (+0.03)	0.857 (0.31-2.35)
10	1.13	1.25 (+0.12)	0.902 (0.61-1.33)

Total absolute CS 23.20% 19.36%. Two-thirds of the decrease in CS rate between 2008 and 2016 is attributable to a drop in the absolute CS rate in Robson groups 1 and 2.

**Table 7:** Robson's 10-Group Classification of a selected group of Flemish women giving birth in 2008 and 2016: relative size of each group in total birth (%).

	2008 (%)	2016 (%)	OR (95% CI)
1	28.6	30	0.934 (0.85-1.02)
2a	13.3	10.6	1.297 (1.14-1.47)
2b	2.1	1.1	1. 833 (1.30-2.61)
3	23.1	26.6	0.829 (0.75-0.91)
4a	11.6	10.9	1.066 (0.94-1.21)
4b	0.5	0.6	0.825 (0.47-1.45)
5	9.3	9.1	1.031 (0.89-1.19)
6	3.1	2.6	1.195 (0.93-1.54)
7	1.7	1.8	0.952 (0.69-1.31)
8	1.7	1.3	1.307 (0.92-1.86)
9	0.2	0.2	0.866 (0.33-2.25)
10	4.8	5.1	0.942 (0.78-1.14)

**Table 8:** Robson's 10-Group Classification of a selected group of Flemish women giving birth in 2008 and 2016: cesarean section per group (%).

	2008 (%)	2016 (%)	OR (95% CI)
1	11.6	8.4	1.425 (1.10-1.85)
2a	22.1	19.3	1.180 (0.87-1.60)
2b	100	100	0
3	2.5	1.3	1.943 (1.01-3.87)
4a	4.1	3.2	1.306 (0.65-2.68)
4b	100	100	0
5	69	64.2	1.242 (0.93-1.66)
6	96.7	97.5	0.743 (0.11-3.91)
7	91.6	94	0.697 (0.17-2.68)
8	57.3	58.3	0.959 (0.46-1.99)
9	90	90.9	0.905 (0.01-78.36)
10	23.7	24.8	0.943 (0.60-1.47)

## Data Availability

The data can be obtained from the agency AZG: “Flemish Agency Care and Health” (http://www.zorg-en-gezondheid.be/cijfers/cijfers-over-geboorte-en-bevalling).
